# Olive Pomace and Melon Bio-Byproducts from the Agribusiness: A Promising Combination for the Sustainable Production of Animal Protein from BSF Larvae

**DOI:** 10.3390/insects16090889

**Published:** 2025-08-26

**Authors:** Carolina Ligeiro, Clarice Silva e Souza, Rafaela Fantatto, Daniel Murta

**Affiliations:** 1 Egas Moniz Center for Interdisciplinary Research (CiiEM), Egas Moniz School of Health & Science, 2829-511 Caparica, Portugal; clarice.souza@entogreen.com (C.S.e.S.); rafaela.fantatto@entogreen.com (R.F.);; 2 Ingredient Odissey SA—Entogreen, 2005-079 Santarém, Portugal

**Keywords:** black soldier fly larvae, bioconversion, *Cucumis melo*, olive pomace, circular economy

## Abstract

The black soldier fly (*Hermetia illucens*) offers a sustainable solution for converting agro-industrial bio-byproducts into high-quality animal protein. This study evaluated two abundant residues from the Santarém region of Portugal, melons and olive pomace, as substrates for larval rearing. Two experimental diets were formulated using these byproducts and compared to a standard Gainesville-based control diet, with all diets adjusted for moisture and protein content. Larval performance was assessed through metrics including individual weight, biomass, feed conversion ratio (FCR) and bioconversion rate. Diets 1 (melon-based) and 2 (melon + 55.2% olive pomace) yielded larval biomass of 1.82 ± 0.17 kg and 1.80 ± 0.11 kg per box, respectively, compared to the control (1.60 ± 0.03 kg). The FCRs were lower in the experimental diets (7.47 ± 0.75 and 7.53 ± 0.49) than in the control (8.42 ± 0.16), and the bioconversion rates were higher (13.48% and 13.31% vs. 11.88%). These results suggest that combining melon and olive pomace improves larval development and conversion efficiency, compared to the literature. This study demonstrates the potential of using local bio-byproducts to enhance insect-based protein production while reducing waste and supporting circular economy practices.

## Simple Summary

Food and agricultural industries generate large amounts of organic byproducts, many of which end up in landfills. This study explored a sustainable solution by using the larvae of the black soldier fly, an insect known for efficiently turning byproducts into animal feed, to recycle two common byproducts in Portugal: melons that were rejected for sale to the final consumer and olive pomace. The goal was to test whether these local byproduct materials could support the growth of healthy larvae that can later be used as a protein-rich ingredient for animal feed. Three different diets were tested, including a standard feed, one with melon, and one combining melon and olive pomace. The results showed that all diets supported good larval development, with the diet combining melon and olive pomace producing strong results in terms of growth and waste reduction. This means that food byproducts such as melons and olive pomace can be transformed into valuable resources. The findings support the idea of turning waste into useful products, reducing environmental impact, and promoting more sustainable farming and food systems.

## 1. Introduction

The Food and Agriculture Organization points out that approximately 14% of global food, equivalent to USD 400 billion annually, is wasted before it even reaches the retail market [1]. Fruits and vegetables account for a large portion of this food waste, especially in industrialized countries, mainly due to post-harvest handling caused by quality standards required by retailers [2].

Fruit byproducts are a rich source of valuable bioactive substances, such as phenolic acids, carotenoids, flavonoids and dietary fiber, among others. Phytochemicals, phytosterols and essential oils are present in large quantities in fruit seeds and peels. In addition, peel, pomace and other parts offer pectin, valuable fibers and minerals [3,4]. Other bio-byproducts cause environmental problems due to their constituents if disposed of incorrectly.

Olive pomace is one of the most abundant agro-industrial bio-byproducts in the Mediterranean region and one of the most challenging, given its high phytotoxicity and potential as a pollutant in some cases, as well as the costs associated with the necessary waste treatments or disposal [5,6]. The European Union (EU), which produces an average of 2 million tons of *Olea europaea* L. annually, is responsible for 63.69% of the world production and is currently the main producer of olive oil [7]. The environmental barrier to increasing production along the olive oil supply chain and the over-intensification of the agricultural phase is the management of the bio-byproducts produced.

For each ton of olive oil produced, the two-phase technique generates 4 tons of pomace, the thick organic bio-byproduct known as “olive pomace”, which consists of fruit pulp, skins, and water from the olive tree vegetation [8,9]. Another highly produced byproduct of the agro-industry is melon (*Cucumis melo* L.). This fruit is cultivated in various parts of the world thanks to its adaptability to many types of soil and temperatures. According to FAOSTAT [10], China was the world’s largest producer of melons (49% of the total, 14 million tons), followed by Turkey and India, although Spain was the world’s largest exporter, producing 600,000 tons, of which 440,000 tons were exported, representing about 20% of total global exports.

Melon bio-byproducts are a potential source of natural food ingredients and can be used to enrich and create new foods with beneficial properties, with a view to promoting health and well-being. To utilize these byproducts and transform them into high-value raw materials for animal nutrition and agriculture, a promising approach to utilize these organic byproducts is bioconversion by larvae of the black soldier fly (*Hermetia illucens* L. *Diptera stratiomyidae*). In most cases, food waste ends up in landfills and is partially recycled through the composting process only at community levels. Sustainable byproduct management can partially offset these problems by achieving significant cost savings when landfilling is replaced by waste-to-wealth approaches [11,12]. The bioconversion rate of black soldier fly larvae (BSFL) is the amount of protein and lipids they can produce from an allocated number of bio-byproducts. The higher the bioconversion rate, the more effectively BSFL transform organic waste into valuable products [13]. The end product of BSF treatment includes both larvae and frass (composed of larval feces, undigested substrate, dead larvae, and exoskeletons). Larvae can be used as a source of protein and animal nutrition, while feces can be used as soil amendment or fertilizer [14].

Similar to other living organisms, BSFL need nutrients to sustain its growth. Therefore, for higher bioconversion performance, BSFL need to feed on organic byproduct rich in digestible nutrients [15,16]. Furthermore, it has been emphasized that BSFL can effectively decompose various types of organic byproducts if they contain an adequate amount of proteins and carbohydrates [17]. However, growth is impaired when BSFL are fed nutrient-deficient organic byproducts. The potential of BSFL to process a wide range of bio-byproducts has gained more attention than that of other fly species, with successful bioconversion reported for diverse substrates such as food waste, brewery spent grains, fruit pulp, and agro-industrial residues [15,18,19,20]. Nowadays, BSF offer a solution to address the challenges of lack of global waste management, unemployment in urban areas, and increasing demand for sustainable animal feed [21]. For this article, two bio-byproducts of greater abundance and prevalence in Portugal were combined to complement each other nutritionally and provide an adequate diet for black soldier fly Larvae.

## 2. Materials and Methods

### 2.1. Bio-Byproducts Production and Chemical Composition

Before the experiment, a questionnaire survey was conducted among different companies in Portugal to identify potential agro-industrial byproducts available in the Santarém region (Table 1).

### 2.2. Diet Formulation

The selection prioritized the valorization of byproducts that, although not currently valued, are obtained during the agro-industrial process. Both producers of melon and olive pomace found this opportunity as a chance to solve logistic and environmental problems, while also recognizing the added value attributed to their byproducts through upcycling their waste in feeding substrate that will be converted into a highly nutritious source for livestock.

The nutritional factor of the byproduct was analyzed to meet the nutritional need of the larvae. Another important factor was the distance between the research unit, Ingredient Odyssey (IO)—EntoGreen R&D, and the agroindustry, as presented in Table 1. The procedures of diet preparation and bioconversion were all carried out at EntoGreen R&D located in Santarém, Portugal. As soon as we received the melons in our R&D unit, they were stored at low temperatures of approximately 4 °C for two weeks until the start of the trial.

To formulate the diets, we used NK-MIX software (v2.8.72), developed by NKMIX Software, Lda. (Peniche, Portugal). This tool applies linear programming to optimize feed formulations based on the nutritional composition and availability of raw materials. The diets were formulated to achieve a moisture content of approximately 70% and similar crude protein content. The latter was adjusted to align with the nutritional profile of the control diet (4.27%), which was based on the Gainesville composite feed (consisting of wheat bran, alfalfa, and maize). Diet 1 was primarily composed of white melon, while Diet 2 consisted of a mixture of melon and olive pomace. The ingredients and nutrient compositions of all three diets are presented in Table 2.

Due to the high moisture content of the main byproducts selected, dry ingredients such as wheat bran were added to ensure the diets had appropriate nutritional value, consistency, and moisture levels. All diets were formulated to maintain the same proportions of protein and moisture, as well as comparable particle sizes and overall homogeneity. The melons were received whole at the R&D unit and required size reduction using an industrial crusher. Once crushed, the melons were mixed according to the specific diet formulation being tested. The mixing process to homogenize each diet lasted for 5 min. In contrast, olive pomace, due to its natural pasty consistency and already small particle size and did not require further grinding.

### 2.3. Bioconversion

After meal preparation, the moisture content was measured. For each dietary combination, 60 cm × 40 cm × 11 cm EntoGreen boxes were filled automatically to a specific weight with six boxes used per treatment. Once filled, a specific number of young larvae were introduced onto the substrate in each box. The boxes were then stored in a controlled warehouse with controlled humidity (60.71 ± 3.64%), temperature (25.56 ± 1.28 °C), and airflow until the bioconversion process was completed.

**Figure 1:**
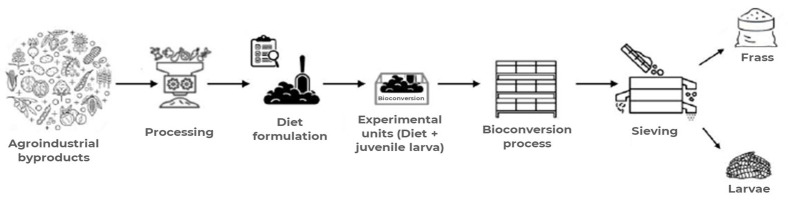
Scheme of the process from byproducts to the separation into larvae and fertilizer.

During the trial, we monitored the substrate temperature by measuring it at three different points within each box. The individual larval weight was assessed by weighing five samples of 10 larvae taken from different locations in each treatment box. After 11 days (once bioconversion was complete and the substrate had reached an appropriate consistency for separation), the contents of each box were sieved to isolate the larvae from the residual frass (insect-derived organic fertilizer). The scheme of these operations is illustrated in Figure 1.

After the sieving process, the larvae were separated, and the following metrics were evaluated: total larval weight per box, individual larval weight (by weighing five samples of 10 larvae per box for each treatment), frass weight per box, and estimated number of larvae per box. The estimated number of larvae per box was calculated using the formula:

(1) $$Number\;of\;larvae=\frac{larval\;biomass\;per\;box\;(g)}{Individual\;larval\;weight\;(g)}\;X\;100$$

The efficiency of BSF larvae in consuming and reducing the tested substrates was determined by calculating substrate reduction (2), feed conversion rate (3), and bioconversion rate (4), following the methodologies described by [22]:

(2) $$Substrate\;reduction\left(\%\right)=\frac{total\;feed\;added-residue\;feed\;after\;treatment}{Total\;feed\;added}\;X\;100$$

(3) $$Feed\;conversion\;rate=\frac{Feed\;added}{Total\;larvae\;biomass}$$

(4) $$Bioconvertion\;rate=\frac{Total\;larvae\;biomass}{Total\;feed\;added}\;X\;100$$

Statistical analysis was conducted using SPSS Statistics software (version 29). After verifying the assumptions for parametric testing, a one-way ANOVA was performed to assess statistical differences between treatment groups. All analyses considered a Type I error probability (α) of 0.05.

## 3. Results

### Larval Performance Parameters, Substrate Consumption, and Reduction Efficiency

**Figure 2:**
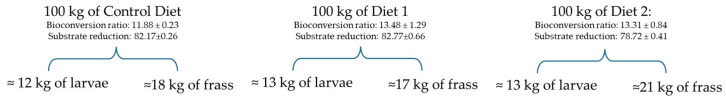
Scheme of the material balance for the conversion of 100 kg of control diet. Diet 1 (melon) and Diet 2 (melon and olive pomace) by BSFL, showing the approximate outputs as larvae and frass.

**Figure 3:**
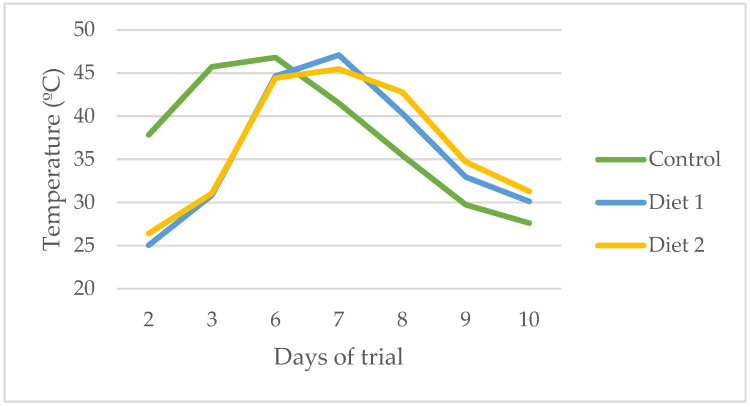
The variation in substrate temperature during biodigestion days.

In this section, we present the results of the bioconversion efficiency analysis for the different experimental diets. Key parameters evaluated include larval individual weight, larval biomass, frass production, number of larvae, substrate reduction, feed conversion ratio (FCR), and bioconversion ratio (Table 3). Additionally, we examined the variation in substrate temperature during the biodigestion process (Figure 2) and the progression of larval weight over time (Figure 3). These results provide insight into how each diet influenced the overall efficiency and dynamics of the bioconversion process.

A significant difference was observed in the amount of frass produced per box, which consequently impacted on the percentage of substrate reduction. Diet 2 generated more frass than the other two diets and, as a result, exhibited a lower substrate reduction percentage. No other parameters showed statistically significant differences. However, there was a noticeable trend toward a slightly higher number of larvae per box (larval biomass) in Diets 1 and 2 compared to the control. In Figure 2, we illustrate the practical application of the bioconversion ratio and substrate reduction, using the example of converting 100 kg of each diet.

Regarding substrate temperature, the control diet began at a higher initial temperature than Diets 1 and 2 (Figure 3). This difference is attributed to the use of heated water in the control mixture, whereas the moisture sources for Diets 1 and 2—olive pomace and melons—were cold. Despite these initial differences, all treatments followed a similar temperature pattern, reaching peak values with only slight differences in timing.

**Figure 4:**
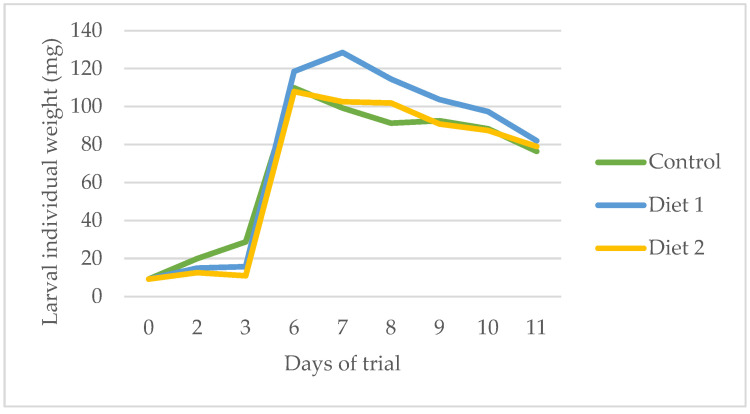
Variation in the weight of the larvae over the days of biodigestion.

The evolution of larval weight over time was similar across all diets (Figure 4). However, larvae fed Diet 1 consistently exhibited higher weights throughout the development period. Despite these differences during growth, the final larval weights were comparable among all diets (Table 3).

## 4. Discussion

The black soldier fly is increasingly recognized as a valuable biological tool for sustainable protein production and the management of organic waste. Its larvae serve as a high-quality feed source for a variety of animal species, with their nutritional profile directly shaped by the substrates they consume [23,24].

Among agro-industrial byproducts, olive pomace is particularly challenging due to its volume, phytotoxicity, and pollution potential [5,6]. Bioconversion using BSF larvae offers a sustainable route to mitigate this issue. In the present study, the incorporation of melon, rich in digestible sugars, appears to have enhanced larval development and overall substrate utilization.

Our findings diverge from those reported by Ramzy et al. [25], where larval weight and survival rates declined with increasing olive pomace concentration. In that study, larvae reared on control diets (without pomace) were approximately 60 mg heavier than those fed diets containing 50% olive pomace. This discrepancy may be attributed to differences in fiber content. While Ramzy et al. [25] used traditionally processed olive pomace with higher lignin levels, our olive pomace had a crude fiber content of 10.3%, and the final fiber content of our Diet 2 (with 55.2% olive pomace) was only 4.27%. These compositional differences may explain the improved outcomes observed in our trials.

It is also important to note that, in this study, the individual larval weight obtained for the control diet was below the reported industrial average of 180 mg [26], which may be attributed to differences in diet composition and rearing conditions

Further supporting this interpretation, Ameixa et al. [27] reported higher FCRs and lower bioconversion rates than those observed in our experiment. Whereas Ameixa et al. [27] documented FCRs of 15.38 (control) and 18.8 (50% olive pomace), our values were significantly lower across all treatments (8.42 ± 0.16 for the control, 7.47 ± 0.75 for Diet 1, and 7.53 ± 0.49 for Diet 2), indicating more efficient substrate conversion by our larvae.

It is important to acknowledge that experimental scales likely influenced these outcomes. Our trials employed larval densities that better reflect industrial conditions, while the studies by Ramzy et al. [25] and Ameixa et al. [27] used only 500 and 100 larvae per treatment, respectively. Smaller-scale studies are more susceptible to environmental fluctuations and may not reliably predict industrial performance [28].

Another distinction lies in the type of substrate used. Previous studies focused solely on olive pomace, whereas our experimental diets also incorporated melon. Melon offers several nutritional advantages, such as being a source of readily available carbohydrates, which likely contributed to the improved larval metrics observed. Although no statistically significant differences in final larval weight were found among treatments, larvae fed Diet 1 (melon-based) exhibited higher individual weights from Day 6 onward, suggesting early developmental benefits.

The type of olive pomace used may also play a crucial role. While most studies utilized dried, full-fat olive pomace, our study employed a moist form, potentially influencing digestibility and nutrient availability. In a large-scale trial by Rodríguez-González et al. [29], higher olive pomace concentrations correlated with increased larval fat content and a gradual growth pattern. However, at higher pomace levels, the FCR values increased significantly compared to the control, unlike our study, where the FCRs remained comparable to or lower than the control, even at 55.2% olive pomace inclusion.

Taken together, our findings indicate that the addition of melon to olive pomace-based diets can mitigate some of the performance drawbacks reported in the literature. The balanced nutritional profile of the combined substrates likely contributed to improved bioconversion and larval growth. These results highlight the importance of substrate composition, processing methods (e.g., dry vs. moist pomace), and experimental scale when interpreting and comparing outcomes across studies.

Lastly, the microbial ecosystem of *H. illucens* may also have played a role. Previous research has shown that the larval gut contains species capable of degrading cellulose, which may improve nutrient assimilation from fiber-rich substrates [30]. This mechanism could help explain the effective performance of larvae fed high-fiber pomace–melon blends in our study.

## 5. Conclusions

To advance the circular economy, minimize agro-industrial byproducts, and add value to materials that would otherwise be discarded, it is essential to adopt ecologically responsible strategies for managing bio-byproducts. This study demonstrates the viability of using BSFL to efficiently bioconvert melon and olive pomace into high-quality animal protein, offering a sustainable alternative for managing these common agro-industrial residues.

The experimental diets formulated with these substrates supported larval development but also achieved high bioconversion efficiency, reinforcing the potential of BSF as a versatile biotechnological tool. Our findings suggest that specific combinations of bio-byproducts, particularly those offering complementary nutritional profiles, may optimize larval performance and waste reduction. This underscores the importance of further research into optimal substrate ratios and processing methods.

Moreover, the value chain can be significantly enhanced by investigating the nutritional quality and potential applications of all outputs from this process—not only larvae as a protein source, but also frass as an organic fertilizer and potential byproducts such as oils and insect-derived feed ingredients.

By integrating agro-industrial waste management with sustainable food production, this approach contributes to a more efficient and resilient food system. Encouraging further research in this area will be crucial to refining practices and unlocking the full potential of BSF-based bioconversion on an industrial scale.

## Figures and Tables

**Table 1:** Survey of potential byproducts present around Santarém, Portugal, as a nutritional source for the diet of the black soldier fly.

Description	Availability	Dry Matter	Crude Protein	Ether Extract	Ash	Crude Fiber	Distance to IO
		%					km
Corn cobbs	September/ October	94.30	1.59	21.10	1.37	28.60	15.00
Grape byproduct (stalk)	September/ October	52.40	3.67	1.20	4.28	23.60	10.00
Wheat bran	All year round	87.94	15.65	4.40	3.20	8.60	40.00
Carrots	All year round	7.70	0.68	0.30	0.47	<1.00	19.10
Mixture of peppers	Mainly in summer	7.30	1.14	0.30	0.41	1.40	19.10
Green peas	Spring	15.10	3.91	0.70	0.55	1.90	19.10
Tomato	Mainly in summer	6.10	1.10	0.40	0.35	1.20	19.10
Green pepper	Mainly in summer	19.80	3.72	3.10	1.23	7.40	19.10
Mixture of peppers with leaves	Mainly in summer	9.00	2.09	5.10	1.31	3.00	19.10
Acorn	Winter	92.20	4.98	9.00	1.82	3.00	50.00
Watermelon	All year round	5.40	0.78	0.30	0.31	<1.00	19.80
Yellow melon	All year round	8.00	0.99	0.50	0.50	1.20	19.80
Green melon	All year round	5.40	0.71	0.30	0.43	<1.00	19.80
White melon	All year round	7.20	0.65	0.30	0.57	<1.00	19.10
Zucchini	All year round	3.60	1.43	0.30	0.34	1.00	19.10
Broccoli	All year round	10.30	3.95	0.50	1.05	1.40	19.10
Olive pomace	From October	23.00	2.28	3.00	1.22	10.30	10.00

IO—Ingredient Odyssey, EntoGreen Research and Development unit.

**Table 2:** Ingredients and nutritional composition of diets used as substrates for rearing the larvae.

			Diets		
			Control	Diet 1	Diet 2
Ingredient Composition	Water	%	66.00		
	Gainesville		34.00	11.60	7.80
	White melon			72.60	21.60
	Olive pomace				55.20
	Wheat bran				15.40
Nutrient Composition	Humidity	%	70.00	70.00	70.00
	Crude protein		4.27	4.27	4.27
	Crude fat		1.01	1.25	2.05
	Crude fiber		3.73	3.84	4.27
	Carbohydrates		1.7	7.3	11.7
	Sugars		5.98	7.77	4.07
	Ashes		2.43	1.99	2.00
	Energy	Kcal	118.06	339.57	174.88

**Table 3:** Parameters of bioconversion efficiency.

Parameters	Control	Diet 1	Diet 2
Larval individual weight (mg)	76.38 ± 9.20	81.95 ± 8.37	78.98 ± 4.26
Larval biomass per box (kg)	1.6 ± 0.03	1.82 ± 0.17	1.80 ± 0.11
Frass per box (kg)	2.41 ± 0.04 ^**a**^	2.33 ± 0.09 ^**a**^	2.87 ± 0.06 ^**b**^
Number of larvae per box	21169 ± 2259	22403 ± 3608	22743 ± 514
Substrate reduction (%)	82.17 ± 0.26 ^**a**^	82.77 ± 0.66 ^**a**^	78.72 ± 0.41 ^**b**^
FCR	8.42 ± 0.16	7.47 ± 0.75	7.53 ± 0.49
Bioconversion ratio (%)	11.88 ± 0.23	13.48 ± 1.29	13.31 ± 0.84

FCR = feed conversion ratio. ^**a**,**b**^ = indicate groups with statistically significant differences between treatments in the parameters (*p* ≤ 0.05).

## Data Availability

Data are available upon request.

## Abbreviations

The following abbreviations are used in this manuscript:

EU	European Union
BSF	Black soldier fly
BSFL	Black soldier fly larvae
IO	Ingredient Odyssey
FCR	Feed conversion ratio

## References

[1] Jenkins W., Tucker M.E., Grim J. (2016). Routledge Handbook of Religion and Ecology.

[2] Porat R., Lichter A., Terry L.A., Harker R., Buzby J. (2018). Postharvest losses of fruit and vegetables during retail and in consumers’ homes: Quantifications, causes, and means of prevention. Postharvest Biol. Technol..

[3] de Albuquerque M.A.C., Levit R., Beres C., Bedani R., LeBlanc A.d.M.d., Saad S.M.I., LeBlanc J.G. (2019). Tropical fruit by-products water extracts as sources of soluble fibres and phenolic compounds with potential antioxidant, anti-inflammatory, and functional properties. J. Funct. Foods.

[4] Islam R., Kamal M., Kabir R., Hasan M., Haque A.R., Hasan S.M.K. (2023). Phenolic compounds and antioxidants activity of banana peel extracts: Testing and optimization of enzyme-assisted conditions. Meas. Food.

[5] Neifar M., Jaouani A., Ayari A., Abid O., Ben Salem H., Boudabous A., Najar T., Ghorbel R.E. (2013). Improving the nutritive value of Olive Cake by solid state cultivation of the medicinal mushroom Fomes fomentarius. Chemosphere.

[6] Roig A., Cayuela M.L., Sánchez-Monedero M.A. (2006). An overview on olive mill wastes and their valorisation methods. Waste Manag..

[7] Kurtoğlu S., Uzundumlu A.S., Gövez E. (2024). Olive Oil Production Forecasts for a Macro Perspective during 2024–2027. Appl. Fruit Sci..

[8] Borja R., Raposo F., Rincón B. (2006). Treatment technologies of liquid and solid wastes from two-phase olive oil mills. Grasas Aceites.

[9] Dermeche S., Nadour M., Larroche C., Moulti-Mati F., Michaud P. (2013). Olive mill wastes: Biochemical characterizations and valorization strategies. Process. Biochem..

[10] FAOSTAT. https://www.fao.org/faostat/es/#data/TCL.

[11] Mohanty S., Saha S., Santra G.H., Kumari A. (2021). Future Perspective of Solid Waste Management Strategy in India. Handbook of Solid Waste Management.

[12] Han Y., Liu J., Xu H. (2022). A comprehensive assessment of the performance of China’s provincial zero-waste cities and impact factor diagnosis. Environ. Impact Assess. Rev..

[13] Siddiqui S.A., Khan S., Farooqi M.Q.U., Singh P., Fernando I., Nagdalian A. (2022). Consumer behavior towards cultured meat: A review since 2014. Appetite.

[14] Klammsteiner T., Turan V., Juárez M.F.-D., Oberegger S., Insam H. (2020). Suitability of Black Soldier Fly Frass as Soil Amendment and Implication for Organic Waste Hygienization. Agronomy.

[15] Kinasih I., Suryani Y., Paujiah E., Ulfa R., Afiyati S., Adawiyah Y., Putra R. (2020). Performance of Black Soldier Fly, *Hermetia illucens*, Larvae during valorization of organic wastes with changing quality. IOP Conf. Ser. Earth Environ. Sci..

[16] Nardiello M., Scieuzo C., Salvia R., Leong S.Y., Kutty R.M. (2020). Characteristic of Fatty Acids Biotransform from *Hermetia illucens* Prepupae Fed with Various Organic Wastes Before Conversion to Methyl Ester Form. IOP Conf. Ser. Mater. Sci. Eng..

[17] Lalander C., Diener S., Zurbrügg C., Vinnerås B. (2019). Effects of feedstock on larval development and process efficiency in waste treatment with black soldier fly (*Hermetia illucens*). J. Clean. Prod..

[18] Taufek N.M., Zulkifli N.F.N.M., Nazri H.A. (2024). Upcycling of Food Waste Generated from the Fresh Market by Utilising Black Soldier Fly Larvae: Influence on Growth, Bioconversion, and Nutritional Composition. J. Environ. Manag..

[19] Magee K., Halstead J., Small R., Young I. (2021). Valorisation of organicwaste by-products using black soldier fly (*Hermetia illucens*) as a bio-convertor. Sustainability.

[20] Arabzadeh G., Delisle-Houde M., Tweddell R.J., Deschamps M.-H., Dorais M., Lebeuf Y., Derome N., Vandenberg G. (2022). Diet Composition Influences Growth Performance, Bioconversion of Black Soldier Fly Larvae: Agronomic Value and In Vitro Biofungicidal Activity of Derived Frass. Agronomy.

[21] Dortmans B.M.A., Egger J., Diener S., Zurbrügg C. (2021). Black Soldier Fly Biowaste Processing—A Step-by-Step Guide.

[22] Diener S., Zurbrügg C., Tockner K. (2009). Conversion of organic material by black soldier fly larvae: Establishing optimal feeding rates. Waste Manag. Res. J. a Sustain. Circ. Econ..

[23] Riekkinen K., Väkeväinen K., Korhonen J. (2022). The Effect of Substrate on the Nutrient Content and Fatty Acid Composition of Edible Insects. Insects.

[24] Maltseva T., Rudoy D., Olshevskaya A., Odabashyan M., Shevchenko V. (2025). Prospects for Using *Hermetia illucens* Larvae in the Diet of Farm Animals: A Review. Online J. Anim. Feed. Res..

[25] Ramzy R.R., El-Dakar M.A., Wang D., Ji H. (2022). Conversion Efficiency of Lignin-Rich Olive Pomace to Produce Nutrient-Rich Insect Biomass by Black Soldier Fly Larvae, *Hermetia illucens*. Waste Biomass-Valorization.

[26] Bonelli M., Bruno D., Caccia S., Sgambetterra G., Cappellozza S., Jucker C., Tettamanti G., Casartelli M. (2019). Structural and functional characterization of *Hermetia illucens* larval midgut. Front. Physiol..

[27] Ameixa O.M.C.C., Pinho M., Domingues M.R., Lillebø A.I., Falabella P. (2023). Bioconversion of olive oil pomace by black soldier fly increases eco-efficiency in solid waste stream reduction producing tailored value-added insect meals. PLoS ONE.

[28] Biasato I., Oddon S.B., Loiotine Z., Resconi A., Gasco L. (2024). Wheat starch processing by-products as rearing substrate for black soldier fly: Does the rearing scale matter?. Animal.

[29] Rodríguez-González E., da Cunha-Borges V., Cantero-Bahillo E., Fornari T., García-Risco M.R., Martin D. (2025). Black soldier fly (*Hermetia illucens*) larvae accumulate bioactive compounds that modulate antioxidant activity when reared with bioactive agrifood by-products. Food Res. Int..

[30] Meneguz M., Schiavone A., Gai F., Dama A., Lussiana C., Renna M., Gasco L. (2018). Effect of rearing substrate on growth performance, waste reduction efficiency and chemical composition of black soldier fly (*Hermetia illucens*) larvae. J. Sci. Food Agric..

